# Stable Episomal Transfectant *Leishmania infantum* Promastigotes Over-Expressing the DEVH1 RNA Helicase Gene Down-Regulate Parasite Survival Genes

**DOI:** 10.3390/pathogens11070761

**Published:** 2022-07-04

**Authors:** Ana Alonso, Jaime Larraga, Francisco Javier Loayza, Enrique Martínez, Basilio Valladares, Vicente Larraga, Pedro José Alcolea

**Affiliations:** 1 Laboratory of Molecular Parasitology and Vaccines, Biological, Immunological, and Chemical Drug Development for Global Health Unit (BICS), Department of Cellular and Molecular Biology, Center for Biological Research Margarita Salas, Spanish Research Council (CIBMS-CSIC), Calle Ramiro de Maeztu 9, 28040 Madrid, Spain; amalonso@cib.csic.es (A.A.); jlarraga@cib.csic.es (J.L.); francisco.loazya@cib.csic.es (F.J.L.); vlarraga@cib.csic.es (V.L.); 2 Department of Obstetrics and Gynecology, Pediatrics, Preventive Medicine and Public Health, Toxicology, Legal and Forensic Medicine and Parasitology, Faculty of Pharmacy, University Institute of Public Health of the Canary Islands (IUETSPC), University of La Laguna (ULL), Avda, Astrofísico Francisco, Sánchez s/n, Campus de Anchieta, 38207 La Laguna, Spain; emartine@ull.es (E.M.); bvallada@ull.es (B.V.)

**Keywords:** DEAD/H RNA helicase, stable episomal transfection, induced over-expression, *Leishmania infantum*, transcriptome

## Abstract

The compartmentalization of untranslated mRNA molecules in granules occurring in many eukaryotic organisms including trypanosomatids involves the formation of complexes between mRNA molecules and RNA-binding proteins (RBPs). The putative ATP-dependent DEAD/H RNA helicase (DEVH1) from *Leishmania infantum* (Kinetoplastida: Trypanosomatidae) is one such proteins. The objective of this research is finding differentially expressed genes in a stable episomal transfectant *L. infantum* promastigote line over-expressing DEVH1 in the stationary phase of growth in axenic culture to get insight into the biological roles of this RNA helicase in the parasite. Interestingly, genes related to parasite survival and virulence factors, such as the hydrophilic surface protein/small hydrophilic endoplasmic reticulum protein (HASP/SHERP) gene cluster, an amastin, and genes related to reactive oxygen species detoxification are down-regulated in DEVH1 transfectant promastigotes.

## 1. Introduction

Leishmaniasis is a vector-borne parasitic disease with an estimated prevalence of 12 million people worldwide. Visceral leishmaniasis (VL) is fatal if left untreated and is responsible for at least 50,000 annual deaths. *Leishmania infantum* (Kinetoplastida: Trypanosomatidae) is the causative pathogen of zoonotic VL in the Mediterranean basin. *L. infantum*–HIV co-infection has been reported in these areas [1,2]. The main reservoirs of *L. infantum* are domestic dogs and wild canids, but hares have also been found to be involved as reservoirs in an active outbreak in central Spain [3,4,5].

Procyclic promastigotes differentiate into metacyclics in the gut of phlebotomine sand fly vectors (Diptera: Psychodidae), which inoculate them into the mammalian host’s dermis. Promastigotes engulfed by phagocytes are able to develop into amastigotes and multiply within phagolysosomes. Eventually, when a sand fly feeds on blood from the infected mammal, amastigotes are released into the gut, and promastigote differentiation begins again, closing the life cycle [6,7,8,9].

In trypanosomatids, protein-coding genes are arranged in long polycistronic gene clusters (PGCs) under the control of a non-canonical promoter constitutively transcribed by RNA polymerase II. Therefore, the steady-state transcript levels are mostly regulated at the post-transcriptional and post-translational levels [10,11,12]. Numerous RNA-binding proteins are encoded in the genomes of these parasites. Transcriptome analysis accounts for steady-state transcript level ratios between the samples compared. However, certain mechanisms of transcriptional control have been reported in these parasites. Chromosome amplification and supernumerary chromosomes are mechanisms for transcription control in *Leishmania* spp. [13]. Differential gene expression rates are relatively low in these parasites [14], but gene expression profiling, mostly performed with transcriptomic approaches, has revealed clues about *Leishmania* differentiation, such as a succession of transient and permanent changes in gene expression during the differentiation of promastigotes to amastigotes [15], the relevance of temperature increase and acidification in this process [16], multiple levels of gene regulation in the parasite [17], the influence of the microenvironment in differentiation and differences between cultured and axenic parasites [18,19,20], genes involved in drug resistance [21], genes essential for promastigote differentiation [18,22,23,24], etc.

One of the gene expression regulation mechanisms observed in *Trypanosoma* spp. and *Leishmania* spp. among other organisms is the compartmentalization of untranslated mRNA molecules in granules together with proteins involved in splicing, transcription, adhesion, and signaling under stress conditions [25,26,27,28,29,30]. mRNAs are regulated in these granules in the cytosol by RNA-binding proteins (RBPs), which form mRNA–protein complexes (mRNPs). RNA helicases are a type of RBP [31] that unwind RNA and displace other RBPs, obtaining energy from ATP [32]. Many of these RNA helicases belong to the SFII superfamily and are classified as DEAD, DEAH, or DEXH according to the sequence of the conserved motif II [33]. Several RNA helicases associate with mRNA in cytoplasmic granules and participate in translation initiation, translation repression, and transcript level decrease through storage or degradation [34]. For example, the DHH1 DEAD-box RNA helicase complexes with mRNA molecules forming granules [26,27,30,35]. One of the RBPs is a putative ATP-dependent DEAD/H RNA helicase (DEVH1) encoded by a gene with the LINF_220021200 identifier in TriTrypDB. This DEVH-box RNA helicase binds to mRNA molecules and accumulates in cytoplasmic granules containing these complexes, especially under stress conditions, as observed in a stable episomal transfectant *L. infantum* promastigote line [36]. These granules contain other proteins involved in splicing, transcription, adhesion, and signaling and are mainly distributed in the cytoplasm periphery [36]. The aim of the present study is to find perturbations in differential gene expression at the mRNA level induced in this transfectant *L. infantum* promastigote line compared to pTEX transfection control promastigotes. This research may improve understanding of the biological functions of this RNA helicase in the parasite.

## 2. Materials and Methods

### 2.1. Parasite Transfection

The *L. infantum* isolate JPCM5 (MCAN/ES/98/LLM−877, zymodeme MON−1) was grown at 26 °C in complete medium containing RPMI 1640 supplemented with L-glutamine and 10% HIFBS. Promastigote samples were harvested at mid-logarithmic phase for transfection. Mid-logarithmic-phase promastigotes were harvested and resuspended at 10^8^ cells/mL in a pre-chilled transfection buffer (21 mM HEPES, 137 mM NaCl, 5 mM KCl, 0.7 mM NaH_2_PO_4_, 6 mM glucose, pH 7.4). Then, aliquots of 0.4 mL of the suspension were poured into 0.2-cm-gap electroporation cuvettes and incubated on ice for 10 min. Meanwhile, aliquots of the pTEX vector [37] and the pTEX-DEVH1 construct [36], previously obtained with the Wizard Plus Maxiprep Kit (Promega, Madison, WI, USA), were adjusted to 1 mg/mL and pre-chilled on ice. Subsequently, 20 μg of DNA were added to the cell suspension, and an electric pulse was applied at 450 V, 500 μF, and 25 Ω with an Electro Cell Manipulator^®^ ECM 630 Precision Pulse^TM^ (BTX^®^, Harvard Apparatus, Holliston, MA, USA). Wild-type parasites suspended in transfection buffer were also electroporated. The cuvettes were immediately placed on ice and chilled for 10 min. Samples were then transferred to culture flasks containing 4 mL of complete medium and grown at 26 °C for 24 h. After 24 h, the selection of stable transfectants was performed under 40 μg/mL G418 selective pressure. Wild-type parasites electroporated in the absence of exogenous DNA were used as selection control under the same selective pressure. Once the stable transfectants were obtained, the cultures were scaled up, harvested in stationary phase (day 7) at 2000× *g* for 10 min, and washed with PBS. These preparations were used for transcriptome analysis. The experiment was performed with all replicate cultures in passage 16. Three biological replicates of the experiment were performed. The pTEX and the pTEX-DEVH1 lines were passaged after the transcriptome analysis. The construct was detected by PCR in passage number 47 (Figure S1).

### 2.2. RNA Isolation, mRNA Amplification, and Indirect Labeling of cDNA with CYANINES

Total RNA extracts were obtained with TRizol reagent (Life Technologies, Carlsbad, CA, USA) following the manufacturer’s instructions and purified with an RNeasy Protect Mini Kit (Qiagen, Hilden, Germany). The quality of the total RNA was assessed by capillary electrophoresis with an Experion RNA HighSens Analysis Kit (Bio-Rad Laboratories, Hercules, CA, USA) according to the manufacturer’s instructions. Then, mRNA was amplified with a MessageAmp II aRNA Amplification Kit (Life Technologies, Carlsbad, CA, USA) as described [28]. The quality of the amplified RNA (aRNA) was assessed by agarose gel electrophoresis. For this purpose, the electrophoresis cell, tray, and comb were rinsed with hydrogen peroxide, and the runs were performed at 5 V/cm in a 1.5% agarose gel prepared with RNase-free water and pre-stained with GelRed Nucleic Acid Gel Stain (Biotium, Hayward, CA, USA) diluted 1:10,000.

Ten micrograms of aRNA were mixed with six micrograms of random hexamer primers (Life Technologies, Carlsbad, CA, USA) to begin the procedure of first-strand aminoallyl-cDNA synthesis. The mixture was denatured at 70 °C for 10 min and immediately chilled on ice. Then, samples were incubated at 46 °C for 3 h with 230 μM dTTP; 340 μM aminoallyl-dUTP; 570 μM (each) dATP, dCTP, and dGTP; 10 μM DTT; and 600 U SuperScript Reverse Transcriptase (Life Technologies) in a final reaction volume of 30 μL. Thereafter, RNA was degraded at 70 °C for 30 min in 100 mM NaOH/10 mM EDTA, and the solution was neutralized with 3 μL of 3 M sodium acetate pH 5.2. Aminoallyl-cDNA was purified with a QiaQuick PCR Purification Kit (Qiagen, Hilden, Germany) following the manufacturer’s instructions except for wash and elution buffers, which were replaced with a phosphate wash buffer (5 mM K_2_HPO_4_, 80% ethanol, pH 8.0) and a phosphate elution buffer (4 mM K_2_HPO_4_). The purified aminoallyl-cDNA samples were completely dried in a vacuum centrifuge, resuspended in 10 μL of water and mixed with 5 μL of Cy3 or Cy5 (samples from pTEX and pTEX-DEVH1 parasites, respectively) monofunctional dye (GE Healthcare, Chalfont Saint Giles, UK) dissolved in DMSO at 12 ng/μL. Next, coupling was allowed at room temperature for 1 h in the dark. Finally, the labeled cDNA samples were purified with a QiaQuick PCR Purification Kit (Qiagen, Hilden, Germany) following the manufacturer’s instructions.

### 2.3. Microarray Hybridization Analysis

Whole-genome shotgun DNA microarrays of *L. infantum* (GEO Accession number GPL6781) were soaked with 0.1% N-lauroylsarcosine in 2XSSC and in 2XSSC, denatured at 95 °C for 3 min, fixed in pre-cooled absolute ethanol, and spin-dried in a minicentrifuge for slides. Afterward, the microarrays were blocked at 42 °C with 60 μL of a buffer containing 2XSSC, 0.3% N-lauroylsarcosine, 60 mM Tris-HCl pH 8.0, 83 ng/mL denatured herring sperm DNA, and 1% BSA using a Hybri-Slip coverslip (Sigma-Aldrich, Buchs, Switzerland) in a hybridization chamber submerged in a water bath for 30 min. Next, labeled cDNA samples were mixed in equimolar amounts of each dye (50 pmol) and incubated at 40 °C with blocked microarrays for 16 h in a blocking solution containing 0.1% BSA, 25 ng/mL poly(T), and 50% deionized formamide. Then, the slides were washed with 2XSSC containing 0.2% SDS at 40 °C, then with 1XSSC and 0.2XSSC at room temperature.

Data acquisition was carried out with a GenePix 4100A instrument (Axon, Foster City, CA, USA). The raw data of local feature background medians were subtracted using GenePix Pro 7.0 software (Axon, Foster City, CA, USA). Normalization was performed with the LOWESS per pin algorithm and statistical inference with the paired Student’s *t*-test by using AlmaZen software (BioAlma, Tres Cantos, Spain). The cutoff values for differential gene expression were defined as follows: (i) fold change F ≥ 1.8 (Cy5/Cy3 ratio if Cy5 > Cy3) or F ≤ −1.8 (−Cy3/Cy5 ratio if Cy3 > Cy5), (ii) total relative fluorescence intensity value >5000 arbitrary fluorescence units, and (iii) *p* < 0.05. Three biological replicates of the experiment were performed.

### 2.4. Identification of Differentially Regulated Genes

The selected clones were isolated, sequenced with the M13-pUC18 universal primers flanking the polylinker of the original genome library, and assembled following an established procedure [38]. In summary, the sequencing reactions of insert ends were set with the m13-pUC18 oligonucleotide primers and run in an ABI Prism^®^ 3730XL Sequencer (Applied Biosystems, Foster City, CA, USA). *L. infantum* JPCM5 chromosomal sequences and annotations were downloaded from TriTrypDB (www.tritrypdb.org, accessed on 17 July 2019). General Feature Format (GFF) files were generated with a Perl script. Read alignments were performed with BLASTN. The boundaries of each clone were defined by pairing forward and reverse sequence reads that fulfilled the following conditions: (i) e-value < 1 × 10^−100^; (ii) convergent orientation between both ends and complementary sequence to different strands of the same chromosome; and (iii) 11 kbp maximum length between the boundaries of each clone. Clones were associated with genes annotated in the *L. infantum* JPCM5 reference genome retrieved from TriTrypDB (www.tritrypdb.org, accessed on 17 July 2019) [39] using a Perl script that excluded 5% of the ORF end sequence that overlapped with the boundaries of the clone. Gene ontology (GO) enrichment analysis was performed with REVIGO [40].

### 2.5. Validation by Real-Time Quantitative RT-PCR (qRT-PCR)

Unlabeled single-stranded cDNA was synthesized as described in Section 2.2 but using a mixture stock of 10 mM of each dNTP. Primers and FAM-NFQ MGB probes (50 nm each; Table S1) were mixed with 1:5 serial dilutions of cDNA samples (10, 2, and 0.4 ng cDNA per reaction) and with TaqMan Universal Master Mix 2X (Life Technologies, Carlsbad, CA, USA) in a final reaction volume of 10 μL. The qRT-PCR reactions were run in a 7900HT Fast Real-Time PCR system using the SDS 4.1. software (Life Technologies, Carlsbad, CA, USA) according to the manufacturer’s instructions. Thermal cycling was as follows: 95 °C for 5 min; 40× (95 °C for 30 s; 60 °C for 1 min, data acquisition). The reference or endogenous control gene was the *L. infantum* gGAPDH. Three experimental replicates and three serial dilutions of each sample were performed. The relative expression values or fold changes were obtained from these data. The raw data and all calculation steps can be found in Supplementary File S1. The standard curve is defined as Ct vs. log m, where m is the total cDNA amount in 10 µL of reaction volume. The standard curve best fit line equation was obtained by the least-squares method. The slope of the serial dilution standard curve was used to calculate the efficiency values (Efficiency = 10^(−1/slope)^). The quantity values (Q) defined as efficiency to the power of –Ct were calculated for all genes including the gGAPDH reference gene. The normalized quantities (*Q_n_*) were obtained by dividing *Q* of the gene of interest by *Q* of gGAPDH. Finally, the qRT-PCR fold changes were obtained as follows:

(1) $$Fold\;change=\{\begin{array}{c} \frac{Q_{n\;pTEX-DEVH1}}{Q_{n\;pTEX}}\;if\;Q_{n\;pTEX-DEVH1}\geq Q_{n}\;\\ -\frac{Q_{n\;pTEX}}{Q_{n\;pTEX-DEVH1}}\;if\;Q_{n\;pTEX-DEVH1}<Q_{n} \end{array}$$

## 3. Results and Discussion

### 3.1. High-Throughput Differential Gene Expression Profiling of Stable Transfectant L. infantum Promastigotes Over-Expressing the DEVH1 Helicase Gene

**Figure 1:**
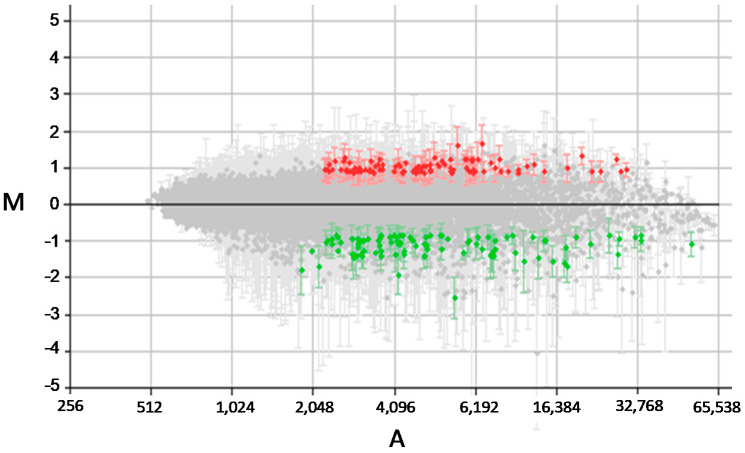
Average M/A scatter plot of three-replicate microarray hybridization analyses. pTEX and pTEX-DEVH1 promastigote expression profiles were compared. M = (log_2_Ri – log_2_Gi) and A = [(log_2_Ri + log_2_Gi)/2], where R and G are, respectively, red (Cy5) and green (Cy3) intensity values. Red spots correspond to selected DNA fragments containing a gene up-regulated at least 1.8 times, and green spots represent those down-regulated at least 1.8 times. The S.D. bars are shown.

Stable episomal transfectant *L. infantum* promastigotes over-expressing the DEVH1 helicase gene (pTEX-DEVH1) were grown until they reached stationary phase in axenic culture. A pTEX transfectant control promastigote line was also generated. The pTEX and pTEX-DEVH1 proliferation rates were similar, reaching the stationary phase on day 7. Total RNA was obtained. Labeled cDNA samples were synthesized prior to microarray hybridization analysis. Three replicates of the experiment were performed. The fold-change value selected as the cutoff for differential gene expression was 1.8 for up-regulation and −1.8 for down-regulation. This criterion was based on the shape of the M/A scatter plot centered in the M = 0 line (Figure 1). The standard deviation (SD) bars contribute to statistical significance evaluated by the Student’s *t*-test. Applying these settings, we found ~100 differentially regulated genes in pTEX-DEVH1 promastigotes compared to pTEX control promastigotes (Figure 1; Table 1). This experiment confirms that the DEVH1 gene is up-regulated (Table 1). 

**Figure 2:**
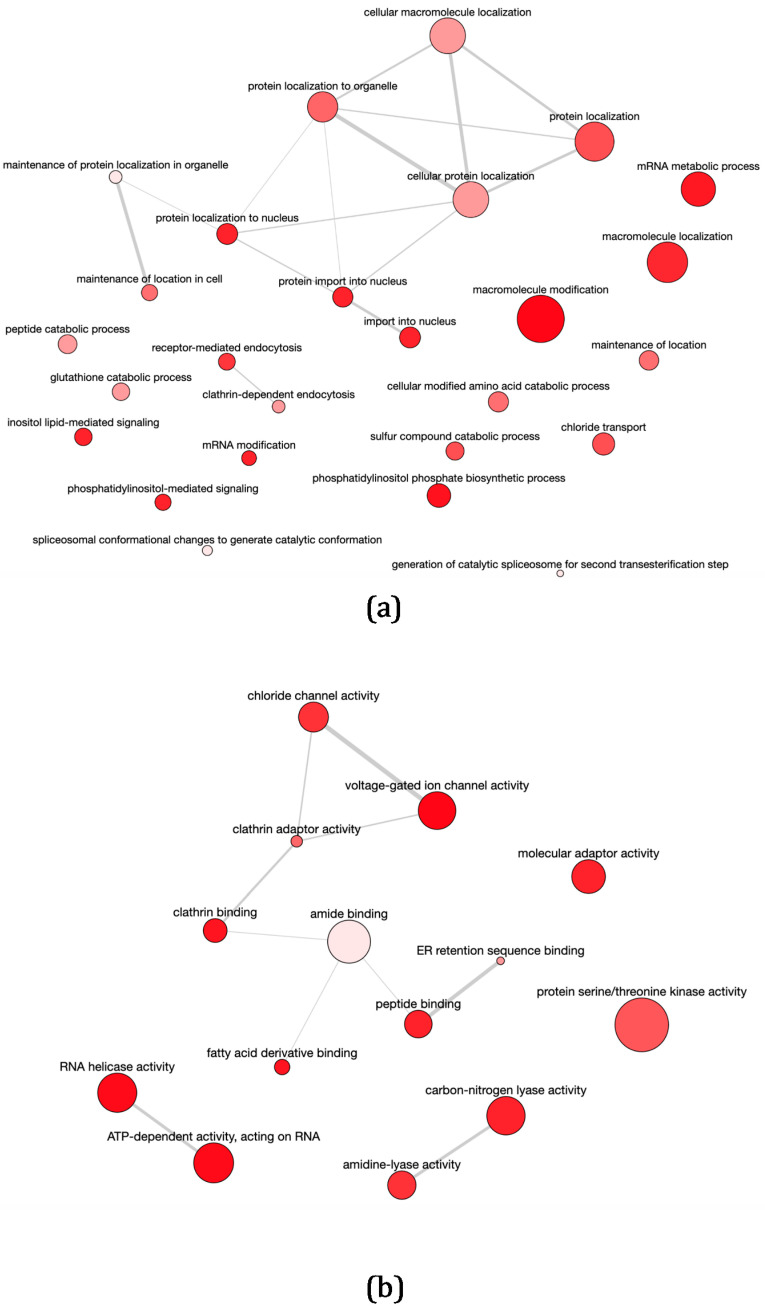
GO enrichment analysis of up-regulated genes in pTEX-DEVH1 promastigotes. Larger spot sizes reflect more general processes and less specific functions. All GO terms represented in this figure are significantly enriched in the subset of up-regulated genes in pTEX-DEVH1 promastigotes. The enrichment analysis is based on the odds ratio calculations. The selected enrichment significance level is α = 0.05. REVIGO allows for the removal of redundancies by applying the neighbor-joining hierarchical clustering algorithm [40]. Functional relationships are represented with gray lines. The thicker the line, the stronger functional relationship. (**a**) Enrichment in GOBP terms. (**b**) Enrichment in GOMF terms.

**Figure 3:**
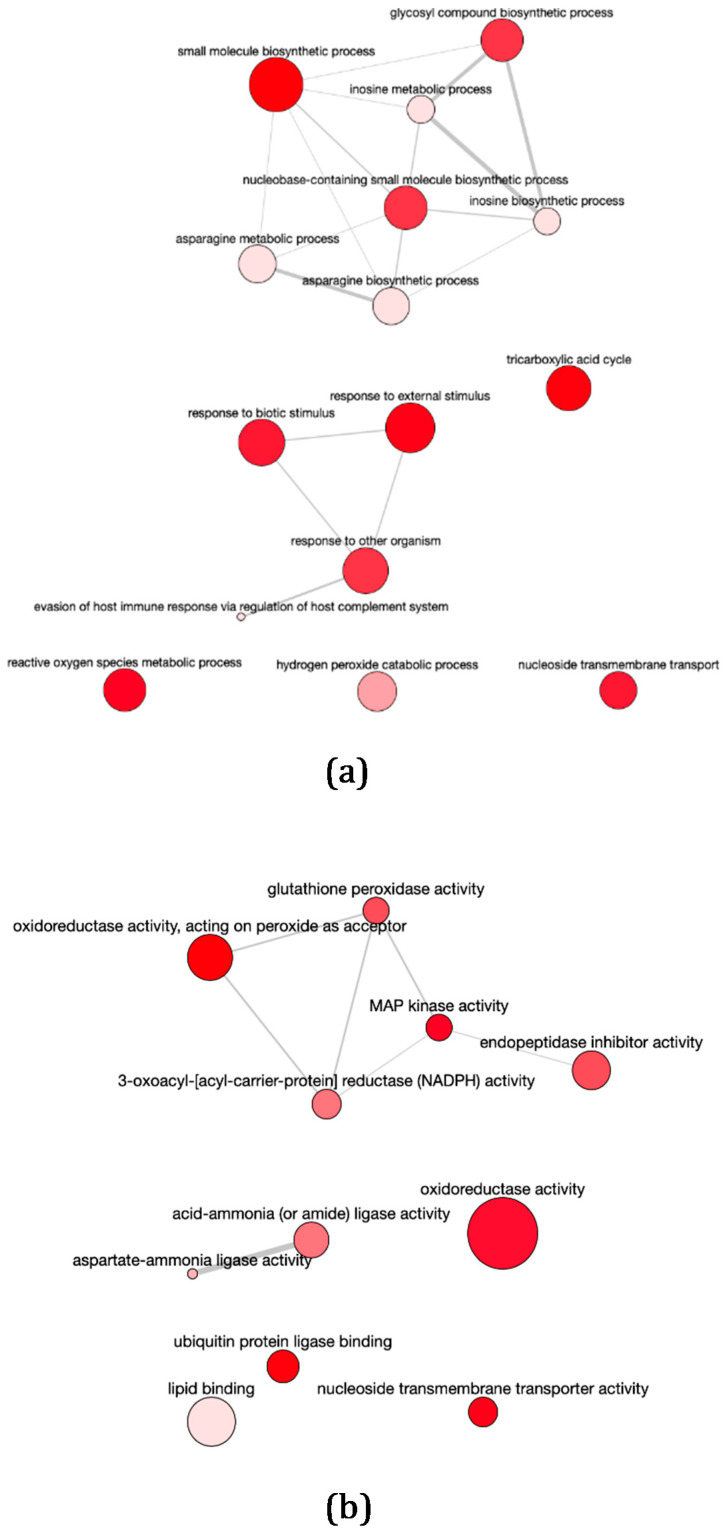
GO enrichment analysis of down-regulated genes in pTEX-DEVH1 promastigotes. Larger spot sizes reflect more general processes and less specific functions. All GO terms represented in this figure are significantly enriched in the subset of down-regulated genes in pTEX-DEVH1 promastigotes. The enrichment analysis is based on the odds ratio calculations. The selected enrichment significance level is α = 0.05. REVIGO allows for the removal of redundancies by applying the neighbor-joining hierarchical clustering algorithm [40]. Functional relationships are represented with gray lines. The thicker the line, the stronger functional relationship. (**a**) Enrichment in GOBP terms. (**b**) Enrichment in GOMF terms.

GO enrichment analyses of Biological Process (GOBP) and Molecular Function (GOMF) terms were performed. The set of genes up-regulated in pTEX-DEVH1 promastigotes is enriched in GOBP terms related with localization and maintenance of proteins and other macromolecules in the cell, metabolism, signaling, and mRNA processing (Figure 2a and Figure S2). The GOMF enrichment analysis indicates that the terms RNA helicase activity and ATP-dependent activity acting on RNA are over-represented in the set of genes up-regulated in pTEX-DEVH1 promastigotes, as well as terms related to transport and intracellular vesicle trafficking (Figure 2a and Figure S2). Significantly over-represented GOBP terms in the set of genes down-regulated in pTEX-DEVH1 promastigotes include response to biotic stimulus; response to other organism; evasion of host immune response via regulation of the host complement system; catabolic processes related with reactive oxygen species (ROS), such as hydrogen peroxide; and nucleotide and amino acid biosynthetic processes (Figure 3a and Figure S3); among others. These terms are related to GOMF terms, such as glutathione peroxidase activity, oxidoreductase activity on peroxide acceptors, other oxidoreductase activities, and ammonia ligase activity (Figure 3a and Figure S3).

### 3.2. Differential Transcript Abundance of Genes Involved in Gene Expression Regulation, Intracellular Signaling, Metabolism, Transport, and Movement in pTEX-DEVH1 L. infantum Promastigotes

An initial exploration of the differentially expressed genes of pTEX-DEVH1 promastigotes and GO enrichment analysis (Figure 2 and Figure 3) suggests an enhancement of processes occurring in undifferentiated, metabolically active promastigotes. Induced DEVH1 over-expression triggers the up-regulation of a 3′−5′ exonuclease gene. The protein product contains an RNase H-like motif (InterPro IPR012337) and a WRN_exo domain (conserved protein domain family database, CDD) related to DNA replication, recombination, and repair. DEVH1 promastigotes also up-regulate several genes involved in gene expression regulation that encode the following protein products: PRP8 homologue U5-snRNA splicing factor; Isy1-like splicing family, involved in splicing optimization (IPR 009360); and the efk−1b isoform of an elongation factor 2-related protein. Conversely, the cullin 2 gene is down-regulated. Cullin 2 contains a winged helix repressor DNA-binding motif, which is related either to transcription repression or helicase activity. Provided that gene expression regulation is mainly post-transcriptional, translational, and post-translational in trypanosomatids (reviewed in [12]), involvement of cullin 2 in helicase activity is more likely in the context of excess DEVH1 in the stable episomal transfectant promastigote line over-expressing the *DEVH1* gene. Protein targeting, modification, and folding may also be influenced by induced DEVH1 over-expression due to up-regulation of the genes encoding a prefolding domain-containing protein, an acyltransferase, a heat shock protein 70 (hsp70), a DnaJ domain-containing protein, a transportin 2-like protein, and an ER lumen targeting protein in pTEX-DEVH1 promastigotes. According to GOBP enrichment analysis (Figure 2a) and protein characterization in model organisms [41], transportins mediate the nuclear import of proteins.

pTEX-DEVH1 promastigotes up-regulate genes encoding for the following proteins involved in transport: glucose transporter GTD2 gene; inosine/guanosine transporter; N-terminal region of chorein (TM vesicle-mediated sorter); right-handed β-helix region—periplasmic copper-binding protein (NosD); voltage-gated chloride channel; an amino acid transporter; and α-adaptin, which is involved in clathrin coating of vesicles. 

Genes related with catabolism have also been found to be up-regulated in pTEX-DEVH1 promastigotes, such as the genes encoding the phosphomannomutase, a glycerol phosphate mutase, the GDP-forming succinyl-CoA ligase β chain, an acyl-CoA-binding protein, and the NADH-cytochrome b5 reductase. On the other hand, genes involved in biosynthesis are down-regulated, including the 3-oxoacyl-ACP reductase (KAR1) and the inosine/guanosine transporter genes.

The comparative transcriptome analysis of pTEX-DEVH1 vs. pTEX control *L. infantum* promastigotes has also revealed that over-expression of this helicase triggers the up-regulation of the genes coding for casein kinase, the phosphatidylinositol 3-kinase 2, the LINF_210006700 serine/threonine protein kinase, an NLI interacting factor-like phosphatase (NLI minimal phosphatase motif, PFAM accession number PF03031), and the conserved hypothetical protein LINF_140020800 (WD40 repeats; InterPro accession number IPR001680). Genes related with the flagellum and the microtubule cytoskeleton dynamics, such as dynein heavy chain and kinesin, are also up-regulated in pTEX-DEVH1 promastigotes. Induced DEVH1 over-expression also triggers up-regulation of the ClanCA, family C2, calpain-like cysteine peptidase gene, which is involved in differentiation, cytoskeleton remodeling, and intracellular signaling processes. On the contrary, a MAP kinase gene, an ADP-ribosylation factor gene, and an NLI interacting factor-like phosphatase (NLI minimal motif) paralog gene are down-regulated.

### 3.3. pTEX-DEVH1 L. infantum Promastigotes Down-Regulate Genes Involved in Parasite Survival

Among the down-regulated genes in pTEX-DEVH1 promastigotes, some encode proteins involved in ROS detoxification, metacyclogenesis, infection, and survival in parasitophorous vacuoles of the host phagocyte. Metacyclic promastigotes up-regulate genes required for the subsequent life cycle stage and for survival within the mammalian host phagocyte, in agreement with the pre-adaptation hypothesis. The GOBP enrichment analysis supports that several genes are involved in resistance to redox and biotic stress, including evasion of the immune response related to the host’s complement system (Figure 3a). This is additional evidence suggesting that induced *DEVH1* gene over-expression achieved in the stable episomal transfectant promastigote line slows down promastigote differentiation and metacyclogenesis. The phosphoglycan β−1,3-galactosyl transferase 4 (β−1,3-GalT4) gene is up-regulated in the pTEX-DEVH1 promastigote line. This gene is involved in lypophosphoglycan (LPG) biosynthesis, which takes place throughout promastigote differentiation. LPG is a major surface molecule of the parasite. LPG is almost completely absent in the amastigote surface but is very abundant in promastigotes (reviewed in [42]).

The glutamine aminotransferase (GLS), the tryparedoxin 1 (TryX), and the type II glutathione peroxidase-like tryparedoxin peroxidase (TrxP) genes are down-regulated in pTEX-DEVH1 promastigotes. GLS yields glutamic acid for glutathione and trypanothione biosynthesis. TrxP is involved ROS detoxification, such as lipid-derived hydroperoxides in trypanosomatids [43].

A concanavalin A-like lectin is down-regulated in pTEX-DEVH1 promastigotes. This gene bears a GOBP term assignment called “evasion of host immune response via regulation of host complement system” (Figure 3a). Promastigotes are subject to complement system clearance [44,45] before a few are able to interact with phagocyte host cells and differentiate to the amastigote stage inside parasitophorous vacuoles.

An amastin-like protein gene (LINF_080011900) up-regulated in amastigotes [24] and in amastigote-like forms induced by temperature increase and acidification [16] is down-regulated in the pTEX-DEVH1 line. Amastin superfamily genes are virulence factors and are supposed to be stage-regulated, increasing expression levels in amastigotes. However, the LINF_080011900 amastin gene is also up-regulated in metacyclic promastigotes in axenic culture [38], supporting that the expression of this gene is enhanced in metacyclic promastigotes. This may be explained by the promastigote pre-adaptation hypothesis [24,46].

**Figure 4:**
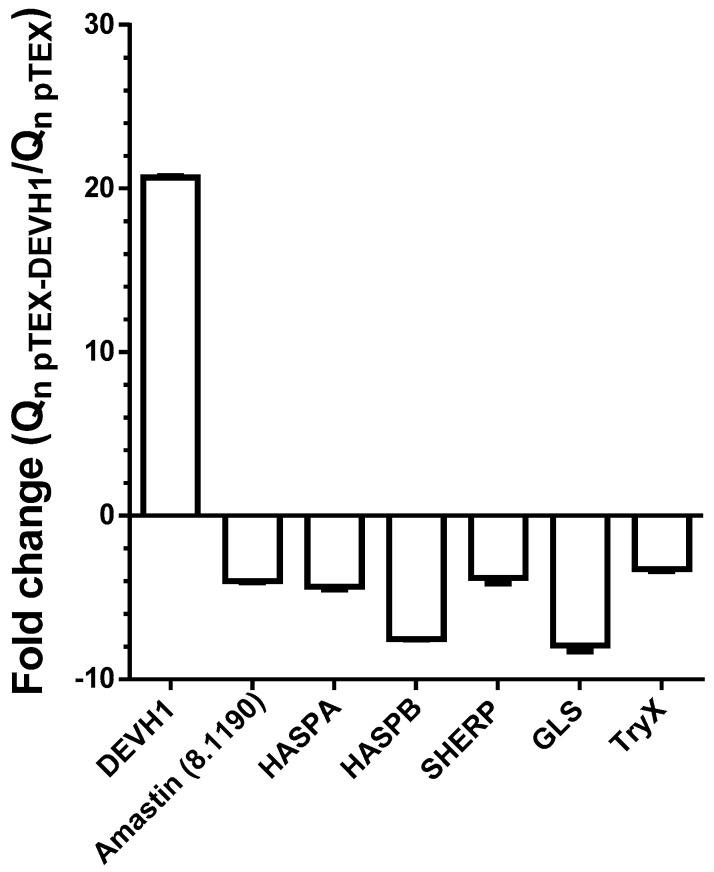
Relative expression levels of DEVH1 and survival genes in pTEX-DEVH1 promastigotes assessed by qRT-PCR. The raw data and the detailed calculations including standard deviations can be found in Supplementary File S1. The fold-changes of the gGAPDH-normalized quantities (Q_n_) are shown. The efficiency values are calculated from the slope of the standard curve using the formula Efficiency = 10^(−1/slope)^. The quantity values are Q = Efficiency^−Ct^ and were calculated for all genes including the gGAPDH reference gene. The normalized quantities are Q_n_ = Q_gene of interest_/Q_gGAPDH_. The fold changes were calculated from the final pTEX-DEVH1 and pTEX quantity values.

The small hydrophilic endoplasmic-reticulum-associated protein (SHERP) and the hydrophilic surface proteins HASPA1, HASPA2, and HASPB have been described as antigens and infective promastigote markers [18,47,48,49,50,51]. The encoding genes are organized in the HASP/SHERP cluster. qRT-PCR analysis using two different calculation methods has confirmed the down-regulation of HASPA, SHERP, and HASPB (Figure 4). HASPA1 and HASPA2 have almost identical sequences (Figure S4) and are not distinguishable by qRT-PCR. Clones containing both copies were identified in the shotgun microarray hybridization analysis (Table 1 and Table S2). The 5′ and 3′ HASPB ends are also practically identical to HASPA1 and HASPA2. Therefore, the TaqMan assay was designed using the inner region of the HASPB nucleotide sequence.

In *Leishmania* spp., the quantitative correlation found between transcript and protein levels has been reported to be low (~25%). However, ~2/3 of all mRNA changes are expected to occur at the protein level as well from a quantitative point of view [18,52,53], which means that the up-regulation, down-regulation, or constant expression is confirmed for both the transcript and the protein for ~67% genes. Among the differentially expressed genes found between pTEX-DEVH1 and pTEX promastigotes, 10 genes characteristic of fully differentiated promastigotes or resistance to oxidative damage are down-regulated, and at least 20 genes that may be related to processes triggered in less differentiated and more metabolically active promastigotes are up-regulated. About ~2/3 of these changes are expected to occur at the protein level too. Furthermore, these genes are grouped in enriched functional categories (Figure 2 and Figure 3).

Elucidating which mRNA molecules of differentially regulated genes bind to the DEVH1 helicase and which transcripts change their steady-state levels by the indirect effect of the DEVH1 helicase is a complex task that may be addressed with the following starting hypothesis. An excess of the DEVH1 helicase may sequester an excess of certain mRNA molecules, making the parasite to re-organize the steady-state transcript levels as a compensation. This may cause a delay in differentiation, as suggested by the down-regulation of the mentioned parasite survival genes (HASP/SHERP cluster, amastin, and redox homeostasis genes). Future experiments with the sand fly *Phlebotomus perniciosus* may elucidate whether the pTEX-DEVH1 line affects parasite competence inside this vector.

## 4. Conclusions

The stable episomal pTEX-DEVH1 *L. infantum* promastigote line down-regulates several genes present in fully differentiated promastigotes or related to ROS detoxification (HASPA1/2, HASPB, SHERP, amastin, concanavalin A-like lectin, GLS, TryX, and TrxP) and up-regulates genes that may be related to processes triggered in less differentiated and more metabolically active promastigotes (e.g., glucose transporter D2, glycerol phosphate mutase, acyl transferase, acyl-CoA-binding protein, amino acid transporter aATP11, phosphoglycan β1→3 galactosyltransferase 4, hsp70, DnaJ domain-containing protein). In summary, 10 genes characteristic of fully differentiated promastigotes including virulence factors or involved in resistance to oxidative damage are down-regulated under DEVH1-induced over-expression, and 20 genes related to growth and differentiation are up-regulated.

## Figures and Tables

**Table 1:** Gene expression profiling of stable DEVH1 *L. infantum* promastigotes. The features described are F (pTEX-DEVH1/pTEX fold change; up-regulation if F ≥ 1.8; down-regulation if F ≤ −1.8); base-two logarithmic scale F and S.D. values; *p*, *p*-value (adjusted by FDR); gene annotation (id and name) in *L. infantum* JPCM5 reference genome (TriTrypDB). Clone identifiers and features can be found in Table S2.

F	log_2_R ± SD	*p*	Id.	Gene Annotation (TriTrypDB)
4.70	2.3 ± 0.4	0.003	LINF_220021200	ATP-dependent DEAD/H RNA helicase, putative
3.04	1.6 ± 0.5	0.035	LINF_300022400	Hypothetical protein, conserved
2.46	1.3 ± 0.2	0.011	LINF_260024900	Hypothetical protein, conserved
2.39	1.3 ± 0.4	0.038	LINF_230022400	Dynein heavy chain, putative
2.38	1.2 ± 0.1	0.002	LINF_360020600	N-terminal region of chorein—a TM vesicle-mediated sorter, putative
2.36	1.2 ± 0.2	0.008	LINF_340018000	Hypothetical protein, conserved
2.34	1.2 ± 0.1	0.004	LINF_140005100	PI3-kinase family—ras-binding domain/Phosphoinositide 3-kinase C2/Phosphoinositide 3-kinase family—accessory domain (PIK domain)/Phosphatidylinositol 3- and 4-kinase—putative
2.32	1.2 ± 0.1	0.004	LINF_340021000	N-terminal region of chorein—a TM vesicle-mediated sorter/Protein of unknown function (DUF1162)—putative
2.32	1.2 ± 0.4	0.043	LINF_320041200	Chloride channel protein, putative
2.31	1.2 ± 0.1	0.002	LINF_250008300	Hypothetical protein, conserved
2.29	1.2 ± 0.1	0.003	LINF_310035300	3’−5’ exonuclease, putative
2.24	1.2 ± 0.3	0.049	LINF_270010200	Calpain-like cysteine peptidase, putative
2.21	1.1 ± 0.2	0.004	LINF_040017400	Hypothetical protein, conserved
2.18	1.1 ± 0.3	0.031	LINF_110011000	Hypothetical protein, conserved
2.15	1.1 ± 0.0	0.000	LINF_330008300	Glucose transporter/membrane transporter D2, putative
2.12	1.1 ± 0.3	0.027	LINF_070005500	Alpha-adaptin-like protein
2.11	1.1 ± 0.3	0.019	LINF_330014800	NLI interacting factor-like phosphatase
2.11	1.1 ± 0.4	0.035	LINF_270028900	WD domain-G-beta repeat, putative
2.10	1.1 ± 0.3	0.027	LINF_160013300	N-terminal region of chorein-A TM vesicle-mediated sorter, putative
2.09	1.1 ± 0.3	0.021	LINF_280011400	ER lumen retaining receptor-like protein
2.06	1.0 ± 0.2	0.010	LINF_360054800	Related to elongation factor−2 kinase efk−1b isoform-like protein
2.06	1.0 ± 0.0	0.001	LINF_360035100	Transportin2-like protein
2.05	1.0 ± 0.3	0.025	LINF_330029900	Glycerol phosphate mutase, putative
2.01	1.0 ± 0.2	0.020	LINF_310041000	Phosphoglycan beta−1,3-galactosyltransferase 4
2.00	1.0 ± 0.4	0.049	LINF_360034600	Hypothetical protein, conserved
1.96	1.0 ± 0.3	0.037	LINF_350031700	Hypothetical protein, conserved
1.96	1.0 ± 0.3	0.035	LINF_360020400	Zn-finger in Ran binding protein and others, putative
1.95	1.0 ± 0.3	0.028	LINF_150005100	Hypothetical protein, conserved
1.95	1.0 ± 0.3	0.032	LINF_350014800	Casein kinase, putative
1.93	0.9 ± 0.1	0.007	LINF_150015300	Hypothetical protein, conserved
1.93	0.9 ± 0.3	0.039	LINF_270023400	Hypothetical protein, conserved
1.92	0.9 ± 0.3	0.033	LINF_220017900	ChaC-like protein, putative
1.91	0.9 ± 0.2	0.019	LINF_350052900	Hsp70 protein, putative
1.91	0.9 ± 0.3	0.025	LINF_240005600	Hypothetical protein, conserved
1.90	0.9 ± 0.2	0.016	LINF_120012700	Hypothetical protein, conserved
1.90	0.9 ± 0.2	0.013	LINF_350047900	Hypothetical protein, conserved
1.88	0.9 ± 0.4	0.049	LINF_060014200	Hypothetical protein, conserved
1.88	0.9 ± 0.3	0.043	LINF_280018500	DnaJ domain-containing protein, putative
1.87	0.9 ± 0.1	0.020	LINF_140020800	Hypothetical protein, conserved
1.87	0.9 ± 0.2	0.010	LINF_350045000	U5 snRNA-associated splicing factor
1.86	0.9 ± 0.1	0.005	LINF_290031800	Acyltransferase, putative
1.86	0.9 ± 0.1	0.007	LINF_310008600	Amino acid transporter aATP11, putative
1.85	0.9 ± 0.3	0.029	LINF_070005100	Isy1-like splicing family—putative
1.84	0.9 ± 0.3	0.032	LINF_170012400	Acyl-CoA-binding protein
1.84	0.9 ± 0.3	0.039	LINF_100018200	Hypothetical protein, conserved
1.84	0.9 ± 0.3	0.045	LINF_360071900	Hypothetical protein, conserved
1.84	0.9 ± 0.3	0.045	LINF_270014400	Right-handed beta helix region/Periplasmic copper-binding protein (NosD), putative
1.84	0.9 ± 0.0	0.000	LINF_340042800	LicD family, putative
1.83	0.9 ± 0.3	0.033	LINF_140013700	Hypothetical protein, conserved
1.83	0.9 ± 0.2	0.017	LINF_170008500	Kinesin motor domain-containing protein, putative
1.81	0.9 ± 0.3	0.036	LINF_230022400	Dynein, heavy chain, putative
1.80	0.8 ± 0.3	0.043	LINF_210006700	Serine/threonine protein kinase, putative
−1.80	−0.8 ± 0.2	0.014	LINF_080011900	Amastin-like protein
−1.80	−0.9 ± 0.3	0.049	LINF_330021300	Glutamine aminotransferase, putative
−1.81	−0.9 ± 0.2	0.025	LINF_310013600	C2 domain protein, putative
−1.82	−0.9 ± 0.2	0.014	LINF_220013600/700	NADH-cytochrome b5 reductase
−1.83	−0.9 ± 0.5	0.044	LINF_060005100	Hypothetical protein, conserved
−1.83	−0.9 ± 0.1	0.007	LINF_190020900	Mitogen-activated protein kinase 4, putative
−1.83	−0.9 ± 0.3	0.029	LINF_120007800	Hypothetical protein, conserved
−1.83	−0.9 ± 0.3	0.028	LINF_120007800	Hypothetical protein, conserved
−1.83	−0.9 ± 0.2	0.019	LINF_270022500	Hypothetical protein, conserved
−1.84	−0.9 ± 0.3	0.042	LINF_290014500	Phytanoyl-CoA dioxygenase (PhyH), putative
−1.85	−0.9 ± 0.3	0.038	LINF_310023200	Hypothetical protein, conserved
−1.87	−0.9 ± 0.1	0.004	LINF_320040300	Hypothetical protein, conserved
−1.88	−0.9 ± 0.0	0.010	LINF_190005300	Histone H2B (H2B)
−1.92	−0.9 ± 0.2	0.016	LINF_230006200	Concanavalin A-like lectin/glucanases superfamily/Beige/BEACH domain-containing protein, putative
−1.93	−0.9 ± 0.4	0.033	LINF_350043500	Hypothetical protein, conserved
−1.94	−1.0 ± 0.0	0.049	LINF_200012700	Tubulin/FtsZ family, putative
−1.95	−1.0 ± 0.0	0.012	LINF_260013100	Type II (glutathione peroxidase-like) tryparedoxin peroxidase
−1.98	−1.0 ± 0.0	0.049	LINF_360061200/300	Vacuolar sorting protein-associated protein-like protein/Aldehyde dehydrogenase, putative
−2.02	−1.0 ± 0.2	0.014	LINF_170008100	Tetratricopeptide repeat—putative
−2.02	−1.0 ± 0.4	0.049	LINF_180018900	Hypothetical protein, conserved
−2.05	−1.0 ± 0.4	0.039	LINF_170005200	Hypothetical protein, conserved
−2.12	−1.1 ± 0.4	0.049	LINF_360045700	Mitogen-activated protein kinase-like
−2.14	−1.1 ± 0.3	0.031	LINF_330016400	Hypothetical protein, conserved
−2.16	−1.1 ± 0.4	0.040	LINF_310022900	Hypothetical protein, conserved
−2.18	−1.1 ± 0.2	0.015	LINF_360037500	GDP-forming succinyl-CoA ligase b chain, putative
−2.20	−1.1 ± 0.3	0.022	LINF_150012500	Ecotin, putative
−2.28	−1.2 ± 0.0	0.046	LINF_160015600	Protein of unknown function (DUF3184), putative
−2.33	−1.2 ± 0.3	0.014	LINF_270032600	3-oxoacyl-ACP reductase, putative (KAR1)
−2.34	−1.2 ± 0.3	0.018	LINF 320037400	Hypothetical protein, conserved
−2.38	−1.2 ± 0.5	0.048	LINF_350007000	NLI interacting factor-like phosphatase—putative
−2.40	−1.2 ± 0.5	0.048	LINF_090014800	Hypothetical protein, conserved
−2.40	−1.3 ± 0.4	0.026	LINF_130007800	Alpha tubulin
−2.44	−1.2 ± 0.0	0.048	LINF_300039100	60S ribosomal protein L9, putative
−2.52	−1.3 ± 0.0	0.033	LINF_120015800/900	Putative integral membrane protein conserved region (DUF2404), putative
−2.53	−1.3 ± 0.5	0.046	LINF_360026000	Inosine-guanosine transporter
−2.56	−1.4 ± 0.5	0.036	LINF_290036400	40S ribosomal protein S19-like protein
−2.57	−1.4 ± 0.7	0.039	LINF_050014100	CPSF A subunit region-containing protein, putative
−2.59	−1.4 ± 0.4	0.028	LINF_290017500	Tryparedoxin 1, putative
−2.65	−1.4 ± 0.5	0.049	LINF_280027900	Cullin 2, putative
−2.67	−1.4 ± 0.1	0.010	LINF_230018600	Hydrophilic surface protein A (HASPA1)
−2.69	−1.4 ± 0.3	0.019	LINF_040008500	ADP ribosylation factor, putative
−2.72	−1.4 ± 0.5	0.046	LINF_360026300	Phosphomannomutase, putative
−2.93	−1.5 ± 0.4	0.023	LINF_300028400	Hypothetical protein, conserved
−3.82	−1.9 ± 0.5	0.021	LINF_230018700	Hydrophilic surface protein B (HASPB)
−3.82	−1.9 ± 0.5	0.021	LINF_230018800	Small hydrophilic endoplasmic-reticulum-associated protein (SHERP)
−3.82	−1.9 ± 0.5	0.021	LINF_230018900	Hydrophilic surface protein A (HASPA2)

## Data Availability

The raw and processed data are available in the Gene Expression Omnibus respository (https://www.ncbi.nlm.nih.gov/geo/query/acc.cgi?acc=GSE202674) (accessed on 30 May 2022).

## Supplementary Materials

The following can be found at https://www.mdpi.com/article/10.3390/pathogens11070761/s1: Figure S1: PCR check of transfected parasites. Figure S2: GO enrichment analysis of up-regulated genes in pTEX-DEVH1 promastigotes. Figure S3: GO enrichment analysis of down-regulated genes in pTEX-DEVH1 promastigotes. Figure S4: Sequence alignment of the HASP/SHERP gene cluster. Table S1: Sequences of primers and FAM-NFQ probes used in qRT-PCR assays. Table S2: Gene expression profiling of pTEX-DEVH1 *L. infantum* promastigotes. Supplementary File S1: qRT-PCR calculations.
