# Supplementary material for: Stable Episomal Transfectant Leishmania infantum Promastigotes Over-Expressing the DEVH1 RNA Helicase Gene Down-Regulate Parasite Survival Genes
Source: Pathogens. 2022 Jul 4;11(7):761. doi: 10.3390/pathogens11070761 (PMC9323391; doi:10.3390/pathogens11070761)
Supplement: Supplementary file 1 [file pathogens-11-00761-s001.zip › pathogens-1755623-supplementary.pdf]

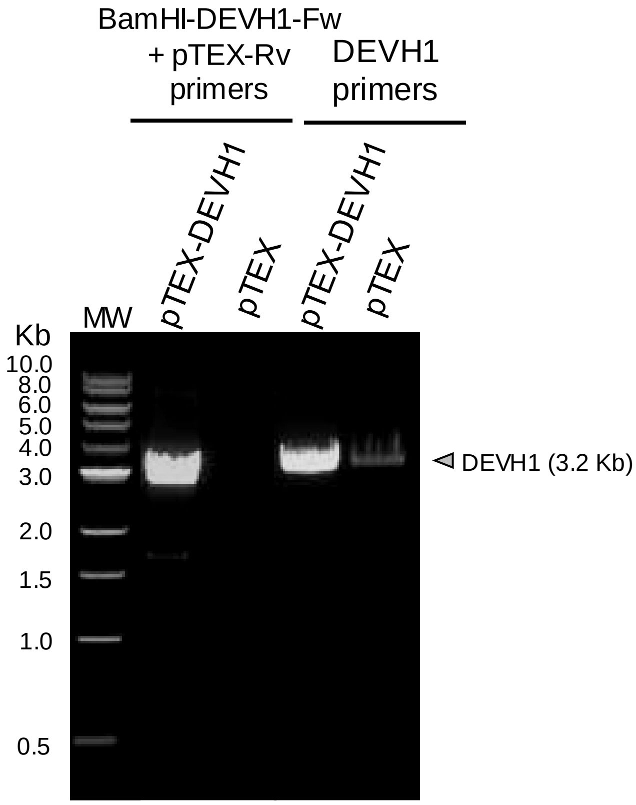

Figure S1. PCR check of transfected parasites. gDNA was extracted from the pTEX-DEVH1 promastigote lines. The DEVH1 gene was amplified using the DEVH1 forward primer including the BamHI restriction site in the 5’ end (BamHI-DEVH1-Fw, GGATCCATGGTACATCGAATTGCA) and a pTEX primer downstream the DEVH1 insert (pTEX-Rv, GCGTTGCCTTGGAGTCGTAAA). The primers for amplification of both chromosomal and exogenous DEVH1 copy were ATGGTACATCGAATTGCA and AAGCTTTTACAGAACTGACATCTC amplifying the whole CDS. No DEVH1 amplificaton with the vector primers is observed in the pTEX line, whereas the DEVH1 chromosomal copy could be amplified in these control promastigotes with the DEVH1 primers.

Biological Process, p<0,05

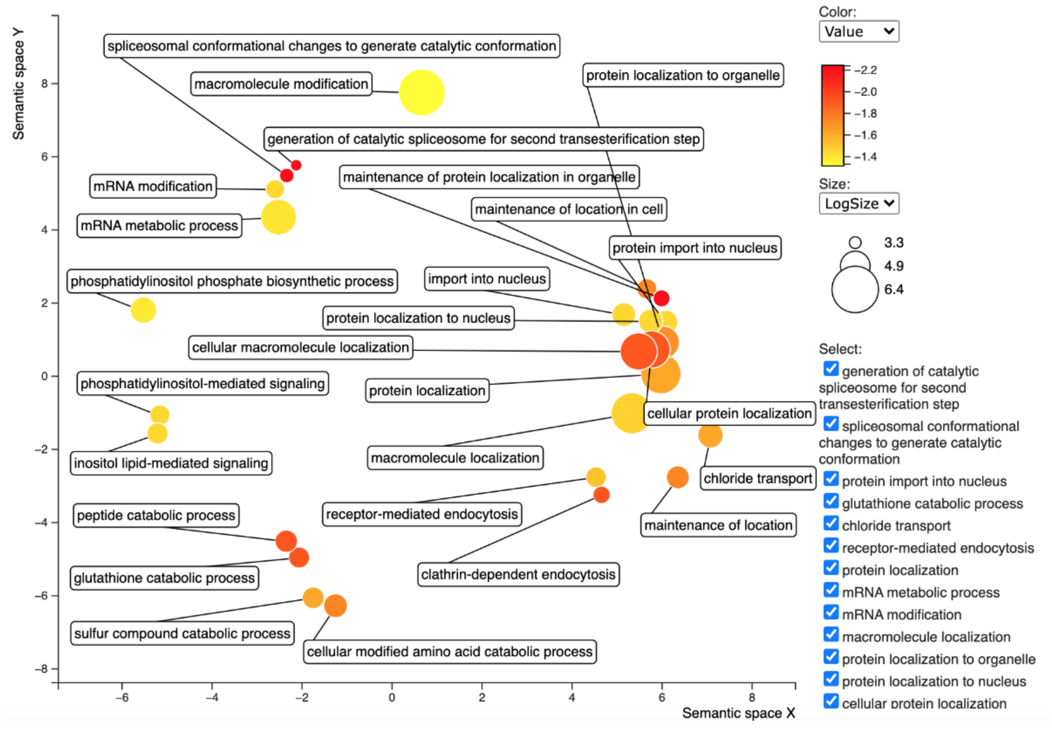

(a)

Molecular Function, p<0,05

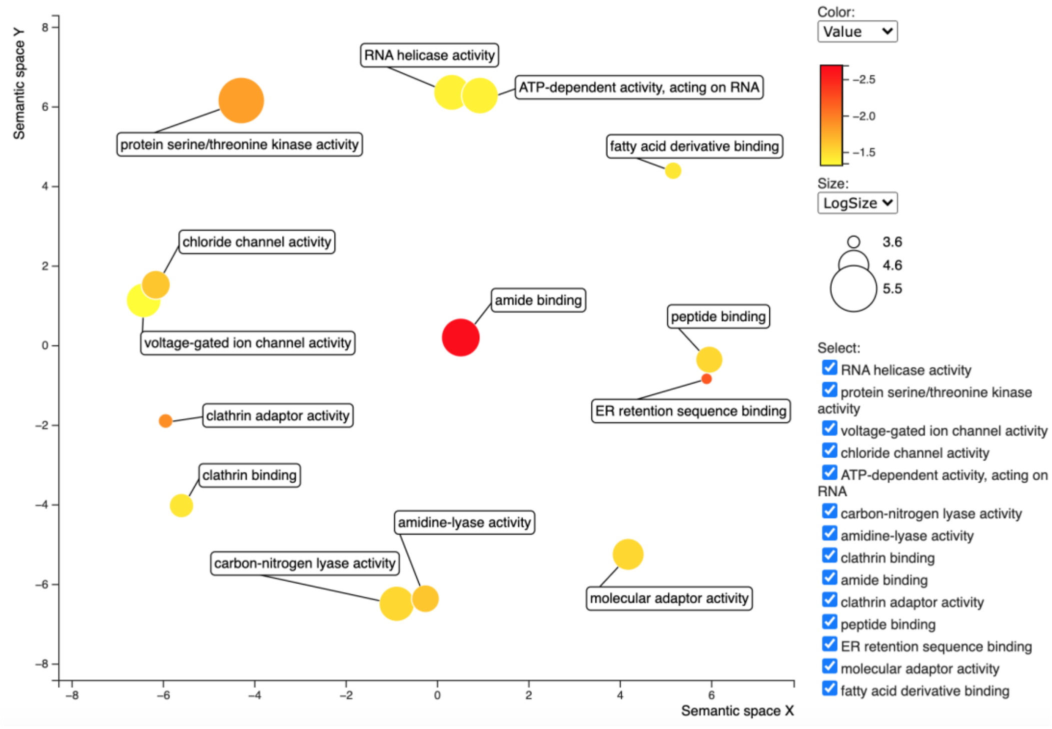

(b)

Figure S2. GO enrichment analysis of up-regulated genes in pTEX-DEVH1 promastigotes. (a) Enrichment in GOBP terms. (b) Enrichment in GOMF terms.

Biological Process p<0,05

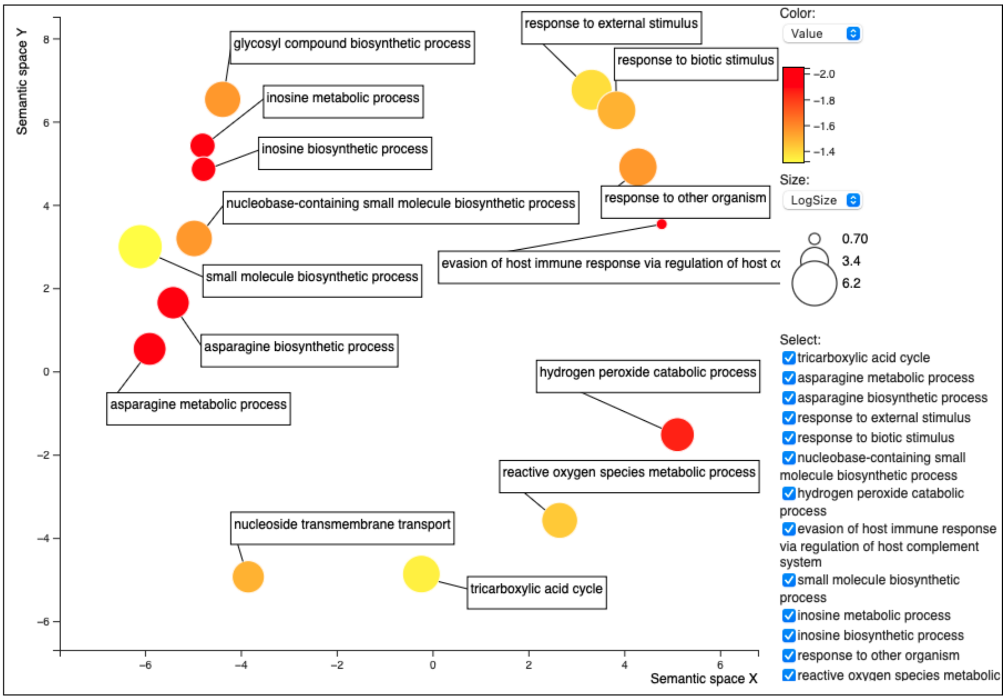

(a)

Molecular Function p>0,05

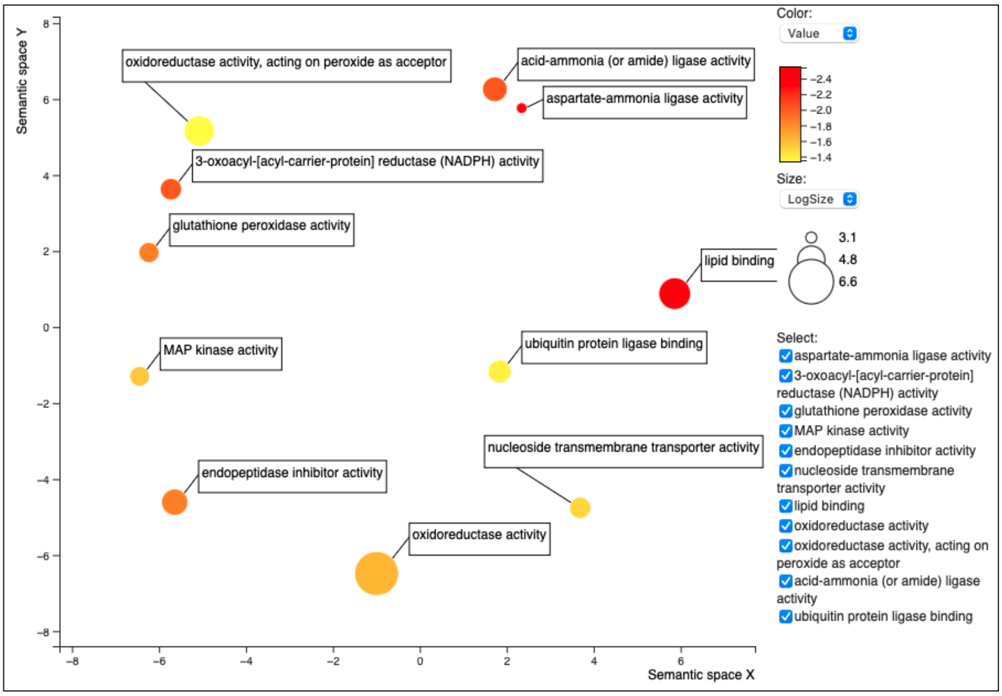

(b)

Figure S3. GO enrichment analysis of down-regulated genes in pTEX-DEVH1 promastigotes. (a) Enrichment in GOBP terms. (b) Enrichment in GOMF terms.

```
SHERP       ---------------------------------------------------------ATGAAC        6
HASPB       ATGGGAGCCTACTGCACGAAGGACTCCGCAAAGGAGCCCCAGAAGCGTGCTGATAACATC        60
HASPA1      ATGGGAAGCTCCTGCACGAAGGACTCCGCAAAGGAGCCCCAGAAGCGTGCTGATAACATC        60
HASPA2      ATGGGAGCCTACTGCACGAAGGACTCCGCAAAGGAGCCCCAGAAGCGTGCTGATAACATC        60
                                                                 *  * *

SHERP       CAGGAGACA-------------------------------------------------AAG       18
HASPB       CATAAAACCACTGAGGCCAATCACAGAGGCGCCGCCGGTGTGCCCCCGAAGCACGCCGGC        120
HASPA1      GATACGACCACTCG----------------------------------------------        74
HASPA2      CATAAAACCACTCG----------------------------------------------        74
             *    **

SHERP       GACCAGATGAACAACGCCGCGGCGGAGACGAGCGACAATGCGCACA--------------        64
HASPB       GGTGCGATGAACGACTCTGCCCCGAAGGAGGATGGCCATACACAGAAAAATGACGGCGAT        180
HASPA1      ------------------------------------------------------------        74
HASPA2      ------------------------------------------------------------        74

SHERP       ------------------------------------------------------------        64
HASPB       GGCCCTAAGGAGGACGGCCGTACACAGAAAAACGACGACGGTGGCCCTAAGGAGGACGGC        240
HASPA1      ------------------------------------------------------------        74
HASPA2      ------------------------------------------------------------        74

SHERP       ----------------------------------------------------ACAAG---        69
HASPB       CATACACAGAAAAATGACGGCGATGGCCCTAAGGAGGACGGCCGTACACAGAAAAATAAC        300
HASPA1      ------------------------------------------------------------        74
HASPA2      ------------------------------------------------------------        74

SHERP       ------------------------------------------------------------        69
HASPB       GGCGATGGCCCTAAGGAGGACGGCCATACACAGAAAAATGACGGCGATGCCCCTAAGGAG        360
HASPA1      ------------------------------------------------------------        74
HASPA2      ------------------------------------------------------------        74

SHERP       ------------------------------------------------------------        69
HASPB       GACGGCCGTACACAGAAAAATGACGGCGATGGCCCTAAGGAGGACGGCCGTACACAGAAA        420
HASPA1      ------------------------------------------------------------        74
HASPA2      ------------------------------------------------------------        74

SHERP       ------------------------------------------------------------        69
HASPB       AATGACGGCGATGGCCCTAAGGAGGACGGCCGTACACAGAAAAATGACGGCGATGGCCCT        480
HASPA1      ------------------------------------------------------------        74
HASPA2      ------------------------------------------------------------        74

SHERP       -------------------------ATCC-------------------------------        73
HASPB       AAGGAGGACGGCCGTACACAGAAAAATGACGGCGATGGCCCTAAGGAGGACGGCCATACA        540
HASPA1      ------------------------------------------------------------        74
HASPA2      ------------------------------------------------------------        74

SHERP       ----------------AGGAGCTGAAGG-------ACGGTGTGAGCAATAAGGCTGCAGA        110
HASPB       CAGAAAAATGACGGCGATGGCCCTAAGGAGGACGGCCGTACACAGAAAAATGACGGCGGT        600
HASPA1      -----------------------------------AAGCGATGAGAAGGACGGCATCCAT        99
HASPA2      -----------------------------------AAGCGATGAGAAGGACGGCATCCAT        99
                                                 *     ** *  * * *  *

SHERP       GGCCCGT-----------------------------------------------------        117
HASPB       GGCCCTAAGGAGGATGAGAATCTGCAGCAAAACGATGGGAATGCGCAGGAGAAGAACGAA        660
HASPA1      GTCCAGGAGAGCGCCGGTCCTGTGCAGGAGAACTTCGGGGATGCGCAGGAGAAGAACGAA        159
HASPA2      GTCCAGGAGAGCGCCGGTCCTGTGCAGGAGAACTTCGGGGATGCGCAGGAGAAGAACGAA        159
            * **

SHERP       ------------------GATGCAGTGGGCAGCACG------------GTTGAGAGCATC        147
HASPB       GATGGACACAACGTGGGGGATGGAGCTAACGGCAATGAGGATGGTAACGATGATCAGCCG        720
HASPA1      GATGGACACAACGTGGGGGATGGAGCTAACGACAATGAGGATGGTAACGATGATCAGCCG        219
HASPA2      GATGGACACAACGTGGGGGATGGAGCTAACGGCAATGAGGATGGTAACGATGATCAGCCG        219
                              **** **     *  **               * ***

SHERP       AAGGACAAGATAAGCGGCGGCTCGTAA 174
HASPB       AAGGAGCAGGTTGCCGGCAACTAG--- 744
HASPA1      AAGGAGCAGGTTGCCGGCAACTAG--- 243
HASPA2      AAGGAGCAGGTTGCCGGCAACTAG--- 243
            *****  ** *   ****  ** *
```

(a)

```
HASPB       MGAYCTKDSAKEPQKRADNIHKTTEANHRGAAGVPPKHAGGAMNDSAPKEDGHTQKNDGD       60
HASPA1      MGSSCTKDSAKEPQKRADNIDTTT------------------------------------       24
HASPA2      MGAYCTKDSAKEPQKRADNIHKTT------------------------------------       24
SHERP       MNQETKDQMNNAAAETSDNAHNKIQELKD---GVS-------------------------       32
            *.   ..:  :   : :** ...

HASPB       GPKEDGRTQKNDDGGPKEDGHTQKNDGDGPKEDGRTQKNNGDGPKEDGHTQKNDGDAPKE       120
HASPA1      ------------------------------------------------------------       24
HASPA2      ------------------------------------------------------------       24
SHERP       ------------------------------------------------------------       32


HASPB       DGRTQKNDGDGPKEDGRTQKNDGDGPKEDGRTQKNDGDGPKEDGRTQKNDGDGPKEDGHT       180
HASPA1      ------------------------------------------------------------       24
HASPA2      ------------------------------------------------------------       24
SHERP       ------------------------------------------------------------       32


HASPB       QKNDGDGPKEDGRTQKNDGG-GPKEDENLQQNDGNAQEKNEDGHNVGDGANGNEDGNDDQ       239
HASPA1      ------------RSDEKDGIHVQESAGPVQENFGDAQEKNEDGHNVGDGANDNEDGNDDQ       72
HASPA2      ------------RSDEKDGIHVQESAGPVQENFGDAQEKNEDGHNVGDGANGNEDGNDDQ       72
SHERP       ------------------------------------NKAAEARDAVGSTV--------ES       48
                                                ::  *  . **. .        :.

HASPB       PKEQVAGN-   247
HASPA1      PKEQVAGN-   80
HASPA2      PKEQVAGN-   80
SHERP       IKDKISGGS   57
             *::::*.
```

(b)

Figure S4. Sequence alignment of the HASP/SHERP gene cluster. (a) Nucleotide sequences. (b) Amino acid sequences of the protein products.

Table S1. Sequences of primers and FAM-NFQ probes used in qRT-PCR assays.

**Primer / probe name**	**Primer /probe sequence**
Amastin-Fw	GCGTCATTATCTACGTCGTC
Amastin-P	CCAGTTCATCGCGTTCTTCTCCGTGCT
Amastin-Rv	ACAGTCAGTCTTTCCACCC
DEVH1-F2	CGCTTGTGTCGTACTTTTCC
DEVH1-P	TCCCGCTCATCACGATCCCTGGTA
DEVH1-Rv	TCCTCCAAAAAAAACTCCCTC
GLS-Fw	TTATTCTCATCAACACGCCTC
GLS-P	CCATCGCATCATTGCCAGACATGTGGGAG
GLS-Rv	CCCACCCAATTTTCCATCC
HASPA1/A2-Fw	CCATGTCCAGGAGAGCGCCGGTC
HASPA1/A2-P	CCATGTCCAGGAGAGCGCCGGTC
HASPA1/A2-Rv	CGTTACCATCCTCATTGCC
HASPB-Fw	AACCACTGAGGCCAATCAC
HASPB-P	CGGCGGTGCGATGAACGACTCTGCCC
HASP-Rv	GCATCGCCGTCATTTTTCTG
SHERP-Fw	TGAACCAGGAGACAAAGGAC
SHERP-P	CGCACAACAAGATCCAGGAGCTGAAGGAC
SHERP-Rv	GCAGCCTTATTGCTCACAC
TryP-Fw	GCCATCCGCTTCTCATCTAC
TryP-P	TGCGGCTTCACCAAGGGCGGCTAC
TryP-Rv	AACTCCTTCACCTCCTCCTC
TryX-Fw	CGAGAAGCATCACAATTCAAAG
TryX-P	CTTTCCATCCCGTTCGAGAAGCGCAAC
TryX-Rv	CGTTCAATCCAATCAGCGTC

Table S2. Gene expression profiling of pTEX-DEVH1 *L. infantum* promastigotes. The features described are: clone number; F (up-regulation if F > 1.8 and down-regulation if F < -1.8); base-two logarithmic scale F and S.D. values; p, p-value (adjusted by FDR); e-value of the boundaries; Def., clone definition according to mapping outcomes a, b or c (Alcolea et al., 2009); annotation id. (in the JPCM5 *L. infantum* genome project sequence); annotated gene function. Clones mapping with more than one differentially regulated gene are not resolved.

Clone	*F*	*$Log_2R \pm SD$*	*p*	*e-value*		*Def.*	*Annotation id.*	*Annotated gene function*
				Fw	*Rv*			
Lin56G10	1.87	0.9 ± 0.1	0.020	3e-33	8e-34	a	LINF_140020800	Hypothetical protein, conserved
Lin60A5	1.84	0.9 ± 0.3	0.032	4e-47	0	a	LINF_170012400	Acyl-CoA-binding protein
Lin70C9	1.86	0.9 ± 0.1	0.005	2e-56	2e-56	a	LINF_290031800	Acyltransferase, putative
Lin84A11	2.10	1.1 ± 0.3	0.027	0	0	a	LINF_160013300	N-terminal region of chorein-A TM vesicle-mediated sorter – putative
Lin87A1	2.09	1.1 ± 0.3	0.021	0	0	a	LINF_280011400	ER lumen retaining receptor-like protein
Lin110B7	1.93	0.9 ± 0.1	0.007	1e-134	1e-136	a	LINF_150015300	Hypothetical protein, conserved
Lin114F6	1.93	0.9 ± 0.3	0.039	2e-50	4e-51	a	LINF_270023400	Hypothetical protein, conserved
Lin131B6	1.84	0.9 ± 0.3	0.039	2e-52	2e-50	a	LINF_100018200	Hypothetical protein, conserved
Lin131G5	1.91	0.9 ± 0.2	0.019	0	0	a	LINF_350052900	Hsp70 protein, putative
Lin132C7	2.01	1.0 ± 0.2	0.020	0	0	b	LINF_310041000	Phosphoglycan beta-1,3-galactosyltransferase 4
Lin134F10	1.81	0.9 ± 0.3	0.036	0	0	a	LINF_230022400	Dynein, heavy chain, putative
Lin150C12	1.88	0.9 ± 0.4	0.049	1e-84	1e-84	a	LINF_060014200	Hypothetical protein, conserved
Lin158E11	2.05	1.0 ± 0.3	0.025	0	0	a	LINF_330029900	Glycerolphosphate mutase, putative
Lin179G6	1.86	0.9 ± 0.1	0.007	1e-131	1e-129	b	LINF_310008600	Amino acid transporter aATP11, putative
Lin186A4	1.90	0.9 ± 0.2	0.016	0	0	a	LINF_120012700	Hypothetical protein, conserved
Lin186B6	2.11	1.1 ± 0.3	0.019	0	0	a	LINF_330014800	NLI interacting factor-like phosphatase
Lin186C12	1.88	0.9 ± 0.3	0.043	7e-62	1e-88	a	LINF_280018500	DnaJ domain containing protein, putative
Lin186D12	1.91	0.9 ± 0.3	0.025	1e-91	1e-91	a	LINF_240005600	Hypothetical protein, conserved
Lin187A12	2.32	1.2 ± 0.1	0.004	0	0	a	LINF_340021000	N-terminal region of Chorein - a TM vesicle-mediated sorter/Protein of unknown function (DUF1162) - putative
Lin187E4	2.34	1.2 ± 0.1	0.004	0	0	a	LINF_140005100	PI3-kinase family - ras-binding domain/Phosphoinositide 3-kinase C2/Phosphoinositide 3-kinase family - accessory domain (PIK domain)/Phosphatidylinositol 3- and 4-kinase – putative
Lin203D10	1.85	0.9 ± 0.3	0.029	0	0	a	LINF_070005100	Isy1-like splicing family – putative
Lin203D12	2.06	1.0 ± 0.2	0.010	1e-178	0	a	LINF_360054800	Related to elongation factor-2 kinase efk-1b isoform-like protein
Lin203G11	1.83	0.9 ± 0.3	0.033	6e-56	1e-52	a	LINF_140013700	Hypothetical protein, conserved
Lin205C2	1.87	0.9 ± 0.2	0.010	1e-44	6e-23	a	LINF_350045000	U5 snRNA-associated splicing factor
Lin211F3	2.15	1.1 ± 0.0	0.000	3e-67	5e-66	b	LINF_330008300	Glucose transporter/membrane transporter D2, putative
Lin213B7	2.11	1.1 ± 0.4	0.035	4e-51	4e-51	a	LINF_270028900	WD domain-G-beta repeat, putative
Lin213D5	2.39	1.3 ± 0.4	0.038	1e-122	1e-124	a	LINF_230022400	Dynein heavy chain, putative
Lin213G12	1.84	0.9 ± 0.3	0.045	1e-26	3e-24	a	LINF_360071900	Hypothetical protein, conserved
Lin214H11	1.92	0.9 ± 0.3	0.033	4e-60	2e-74	b	LINF_220017900	ChaC-like protein, putative
Lin215C11	1.84	0.9 ± 0.3	0.045	1e-150	0	a	LINF_270014400	Right handed beta helix region/Periplasmic copper-binding protein (NosD) – putative
Lin215E6	1.83	0.9 ± 0.2	0.017	0	0	a	LINF_170008500	Kinesin motor domain containing protein, putative
Lin218H1	2.29	1.2 ± 0.1	0.003	0	0	a	LINF_310035300	3'-5' exonuclease – putative
Lin220E5	2.24	1.2 ± 0.3	0.049	0	0	a	LINF_270010200	Calpain-like cysteine peptidase, putative
Lin220H5	1.80	0.8 ± 0.3	0.043	2e-58	1e-63	a	LINF_210006700	Serine/threonine protein kinase, putative
Lin221A3	2.36	1.2 ± 0.2	0.008	5e-69	1e-156	a	LINF_340018000	Hypothetical protein, conserved
Lin221A4	2.38	1.2 ± 0.1	0.002	1e-134	1e-130	a	LINF_360020600	N-terminal region of Chorein - a TM vesicle-mediated sorter – putative
Lin224A11	2.00	1.0 ± 0.4	0.049	0	0	a	LINF_360034600	Hypothetical protein, conserved
Lin225A8	2.06	1.0 ± 0.0	0.001	0	0	a	LINF_360035100	Transportin2-like protein
Lin232A10	2.21	1.1 ± 0.2	0.004	0	0	a	LINF_040017400	Hypothetical protein, conserved
Lin237A10	2.46	1.3 ± 0.2	0.011	0	0	a	LINF_260024900	Hypothetical protein, conserved
Lin237B7	1.96	1.0 ± 0.3	0.037	0	0	a	LINF_350031700	Hypothetical protein, conserved
Lin237E9	1.95	1.0 ± 0.3	0.028	0	0	a	LINF_150005100	Hypothetical protein, conserved
Lin239C5	4.70	2.3 ± 0.4	0.003	0	0	a	LINF_220021200	ATP-dependent DEAD/H RNA helicase, putative
Lin255C8	1.90	0.9 ± 0.2	0.013	1e-117	0	a	LINF_350047900	Hypothetical protein, conserved

Lin258B5	1.95	1.0 ± 0.3	0.032	1e-44	1e-44	b	LINF_350014800	Casein kinase, putative
Lin258B6	2.12	1.1 ± 0.3	0.027	0	0	a	LINF_070005500	Alpha-adaptin-like protein
Lin281G2	1.84	0.9 ± 0.0	0.000	3e-42	3e-42	a	LINF_340042800	LicD family, putative
Lin282H12	1.96	1.0 ± 0.3	0.035	0	0	b	LINF_360020400	Zn-finger in Ran binding protein and others, putative
Lin285E2	2.31	1.2 ± 0.1	0.002	0	0	a	LINF_250008300	Hypothetical protein, conserved
Lin285H5	2.18	1.1 ± 0.3	0.031	0	0	a	LINF_110011000	Hypothetical protein, conserved
Lin289C6	3.04	1.6 ± 0.5	0.035	1e-107	2e-96	a	LINF_300022400	Hypothetical protein, conserved
Lin301F6	2.32	1.2 ± 0.4	0.043	0	0	a	LINF_320041200	Chloride channel protein, putative
Lin32A8	-2.28	-1.2 ± 0.0	0.046	0	0	b	LINF_160015600	Protein of unknown function (DUF3184), putative
Lin53F1	-2.14	-1.1 ± 0.3	0.031	0	0	b	LINF_330016400	Hypothetical protein, conserved
Lin59C3	-2.20	-1.1 ± 0.3	0.022	9e-43	1e-38	a	LINF_150012500	Ecotin, putative
Lin62C3	-2.67	-1.4 ± 0.2	0.010	0	0	a	LINF_230018600	Hydrophilic surface protein A (HASPA1)
Lin73E8	-2.44	-1.2 ± 0.0	0.048	0	0	a	LINF_300039100	60S ribosomal protein L9, putative
Lin77H8	-1.80	-0.8 ± 0.2	0.014	0	0	b	LINF_080011900	Amastin-like protein
Lin77H12	-1.98	-1.0 ± 0.0	0.049	0	0	b	LINF_360061200/ 300	Vacuolar sorting protein-associated protein-like protein/ Aldehyde dehydrogenase, putative
Lin106B3	-2.18	-1.1 ± 0.2	0.015	0	0	a	LINF_360037500	GDP-forming succinyl-CoA ligase β chain, putative
Lin109B8	-2.53	-1.3 ± 0.5	0.046	0	0	b	LINF_360026000	Inosine-guanosine transporter
Lin123B1	-2.12	-1.1 ± 0.4	0.049	0	0	a	LINF_360045700	Mitogen-activated protein kinase-like
Lin123C12	-2.38	-1.2 ± 0.5	0.048	0	0	b	LINF_350007000	NLI interacting factor-like phosphatase – putative
Lin130D8	-2.40	-1.2 ± 0.5	0.048	0	0	b	LINF_090014800	Hypothetical protein, conserved
Lin136G4	-2.52	-1.3 ± 0.0	0.033	0	0	a	LINF_120015800/ 900	Putative integral membrane protein conserved region (DUF2404), putative
Lin142D11	-2.40	-1.3 ± 0.4	0.026	0	0	a	LINF_130007800	Alpha tubulin
Lin158F5	-1.85	-0.9 ± 0.3	0.038	0	0	b	LINF_310023200	Hypothetical protein, conserved
Lin162C12	-2.57	-1.4 ± 0.7	0.039	0	0	b	LINF_050014100	CPSF A subunit region containing protein, putative
Lin163A7	-3.82	-1.9 ± 0.5	0.021	0	2e-170	b	LINF_230018700	Hydrophilic surface protein (HASPB)
Lin163A7	-3.82	-1.9 ± 0.5	0.021	0	2e-170	b	LINF_230018800	Small hydrophilic endoplasmic reticulum-associated protein (SHERP)
Lin163A7	-3.82	-1.9 ± 0.5	0.021	0	2e-170	b	LINF_230018900	Hydrophilic surface protein A (HASPA2)
Lin163A8	-2.33	-1.2 ± 0.3	0.014	1e-134	1e-114	b	LINF_270032600	3-oxoacyl-ACP reductase, putative (KAR1)
Lin164H4	-2.65	-1.4 ± 0.5	0.049	7e-90	4e-88	a	LINF_280027900	Cullin 2, putative
Lin166H9	-1.84	-0.9 ± 0.3	0.042	0	0	b	LINF_290014500	Phytanoyl-CoA dioxygenase (PhyH), putative
Lin166H10	-2.93	-1.5 ± 0.4	0.023	0	0	a	LINF_300028400	Hypothetical protein, conserved
Lin192G3	-1.95	-1.0 ± 0.0	0.012	6e-47	2e-43	b	LINF_260013100	Type II (glutathione peroxidase-like) tryparedoxin peroxidase
							LINF_260013200	Aspartate-ammonia ligase, putative
Lin205H2	-1.92	-0.9 ± 0.2	0.016	1e-180	0	b	LINF_230006200	Concanavalin A-like lectin/glucanases superfamily/Beige/BEACH domain containing protein, putative
Lin207G10	-1.83	-0.9 ± 0.5	0.044	0	0	b	LINF_060005100	Hypothetical protein, conserved
Lin208G9*	-1.88	-0.9 ± 0.0	0.010	2e-59	0	b	LINF_190005300	Histone H2B (H2B)
Lin209F5	-1.82	-0.9 ± 0.2	0.014	0	0	a	LINF_220013600/ 700	NADH-cytochrome b5 reductase
Lin209G3	-2.02	-1.0 ± 0.2	0.014	0	0	c	LINF_170008100	Tetratricopeptide repeat – putative
Lin210C2	-1.81	-0.9 ± 0.2	0.025	0	0	b	LINF_310013600	C2 domain protein, putative
Lin224E3	-1.83	-0.9 ± 0.1	0.007	0	0	a	LINF_190020900	Mitogen-activated protein kinase 4, putative
Lin225C1	-2.59	-1.4 ± 0.4	0.028	2e-71	3e-83	b	LINF_290017500	Tryparedoxin 1, putative
Lin230C6	-2.72	-1.4 ± 0.5	0.046	0	0	b	LINF_360026300	Phosphomannomutase, putative
Lin230E6	-1.83	-0.9 ± 0.3	0.029	0	0	b	LINF120007800	Hypothetical protein, conserved
Lin238G11	-1.83	-0.9 ± 0.3	0.028	6e-158	0	b	LINF120007800	Hypothetical protein, conserved
Lin239G8	-2.34	-1.2 ± 0.3	0.018	0	0	b	LINF320037400	Hypothetical protein, conserved
Lin241D5	-2.05	-1.0 ± 0.4	0.039	0	0	a	LINF_170005200	Hypothetical protein, conserved
Lin265C7	-1.93	-0.9 ± 0.4	0.033	0	0	a	LINF_350043500	Hypothetical protein, conserved
Lin269B5	-2.56	-1.4 ± 0.5	0.036	0	0	b	LINF_290036400	40S ribosomal protein S19-like protein
Lin269D6	-1.87	-0.9 ± 0.1	0.004	1e-98	0	a	LINF_320040300	Hypothetical protein, conserved
Lin294C9	-2.69	-1.4 ± 0.3	0.019	0	1e-82	a	LINF_040008500	ADP ribosylation factor, putative
Lin295F4	-1.94	-1.0 ± 0.0	0.049	0	0	b	LINF_200012700	Tubulin/FtsZ family, putative
Lin298A4	-1.83	-0.9 ± 0.2	0.019	1e-165	0	b	LINF_270022500	Hypothetical protein, conserved
Lin298B12	-1.80	-0.9 ± 0.3	0.049	0	0	b	LINF_330021300	Glutamine aminotransferase, putative
Lin298E9	-2.02	-1.0 ± 0.4	0.049	0	0	b	LINF_180018900	Hypothetical protein, conserved
Lin300G1	-2.16	-1.1 ± 0.4	0.040	1e-100	2e-19	b	LINF_310022900	Hypothetical protein, conserved
